# 3D Printed Solutions for Spheroid Engineering and Cancer Research

**DOI:** 10.3390/ijms23158188

**Published:** 2022-07-25

**Authors:** Tobias Butelmann, Yawei Gu, Aijun Li, Fabian Tribukait-Riemenschneider, Julius Hoffmann, Amin Molazem, Ellen Jaeger, Diana Pellegrini, Aurelien Forget, V. Prasad Shastri

**Affiliations:** 1 Institute for Macromolecular Chemistry, University of Freiburg, 79104 Freiburg, Germany; tobias.butelmann@makro.uni-freiburg.de (T.B.); yawei.gu@makro.uni-freiburg.de (Y.G.); aijun.li@makro.uni-freiburg.de (A.L.); fabian.riemenschneider@makro.uni-freiburg.de (F.T.-R.); varusgaius@gmail.com (J.H.); molazem_amin@yahoo.de (A.M.); ellen.j.jaeger@web.de (E.J.); dianapellegrini34@gmail.com (D.P.); aurelien.forget@makro.uni-freiburg.de (A.F.); 2 BIOSS Centre for Biological Signalling Studies, University of Freiburg, 79104 Freiburg, Germany

**Keywords:** tumor spheroids, hanging drop device, 3D printing, bioprinting, automation

## Abstract

In multicellular organisms, cells are organized in a 3-dimensional framework and this is essential for organogenesis and tissue morphogenesis. Systems to recapitulate 3D cell growth are therefore vital for understanding development and cancer biology. Cells organized in 3D environments can evolve certain phenotypic traits valuable to physiologically relevant models that cannot be accessed in 2D culture. Cellular spheroids constitute an important aspect of in vitro tumor biology and they are usually prepared using the hanging drop method. Here a 3D printed approach is demonstrated to fabricate bespoke hanging drop devices for the culture of tumor cells. The design attributes of the hanging drop device take into account the need for high-throughput, high efficacy in spheroid formation, and automation. Specifically, in this study, custom-fit, modularized hanging drop devices comprising of inserts (Q-serts) were designed and fabricated using fused filament deposition (FFD). The utility of the Q-serts in the engineering of unicellular and multicellular spheroids-synthetic tumor microenvironment mimics (STEMs)—was established using human (cancer) cells. The culture of spheroids was automated using a pipetting robot and bioprinted using a custom bioink based on carboxylated agarose to simulate a tumor microenvironment (TME). The spheroids were characterized using light microscopy and histology. They showed good morphological and structural integrity and had high viability throughout the entire workflow. The systems and workflow presented here represent a user-focused 3D printing-driven spheroid culture platform which can be reliably reproduced in any research environment and scaled to- and on-demand. The standardization of spheroid preparation, handling, and culture should eliminate user-dependent variables, and have a positive impact on translational research to enable direct comparison of scientific findings.

## 1. Introduction

According to the World Health Organization, cancer remains the first or second leading cause of premature death in 134 of 183 countries with lung, breast, and colorectal cancers being the most prominent causes [1]. Cancer places a huge burden on health care systems and contributes to diminished quality of life for the patient and caregivers. One of the primary objectives of cancer research is to unravel the origins of cancer and identify new therapeutic targets. In the classic review by Hanahan and Weinberg on the hallmarks of cancer, in addition to identifying major characteristics of malignant neoplasms, they also proposed significant roles for stromal cells in tumor development and invasion [2,3]. The impact of different cells (e.g., lymphocytes, cancer associated fibroblasts, endothelial cells) and the role of an extracellular matrix (ECM) and its changes during the progression of cancer growth is now well-recognized, and this collectively represents the tumor microenvironment (TME). Towards emulating TME, 3D cell culture (3DCC) models are considered superior to 2D culture as in a 3D matrix, cells experience physiologically authentic interactions and microenvironments [4,5,6]. 3DCC approaches can be divided into scaffold-free and scaffold-based methods and both approaches have gained importance since the 1970s. The scaffold-free method offers the following advantages: the self-aggregation and interaction of cells in 3D, endogenous molecular and structural gradients, and the secretion of ECM [7,8]. As a result, 3DCC are extensively used in topics covering multicellular spheroids, drug research, and organoid culture [9,10,11]. Scaffold-based cell culturing on the other hand, is a method that involves the association of cells with a 3D matrix thus mimicking the interaction of cells with the ECM. An important variable in this approach is the biomaterial serving as the scaffold. In this respect, hydrogels have been regarded as ideal materials to provide the cells with physical support, as they can be extensively tailored to present specific mechanical, chemical, and biological cues, thus replicating important ECM attributes such as hydration, stiffness and biofunctionality [12,13]. Several biopolymers are capable of forming hydrogels and among them collagen type-I, hyaluronic acid (HA), and alginate are common examples used in cell biology [14,15].

In recent years, with advances in 3D bioprinting (3DBP), it is now possible to precisely reconstitute the geometry of tissues in vitro [16,17,18,19,20]. Similar to scaffold-based 3DCC, hydrogels are the most common carriers and serve a support function in 3DBP and are referred to as bioinks [21], in the sense they are the medium through which biological information is scribed. In combination, 3DCC and 3DBP can provide a novel pathway to building tumor models in vitro that can mimic the attributes of in vivo TME with respect to tethered and soluble biological signals, ECM mechanics, and cell composition. For example, tumors present cellular and ECM spatial heterogeneity and the relation between the tumor and tumor-adjacent tissues play pivotal roles since they do not only reflect the growth pattern of tumor cells but also determine the invasion patterns [22]. Such a complex juxtaposition of information can be readily replicated using 3DBP.

**Figure 1:**
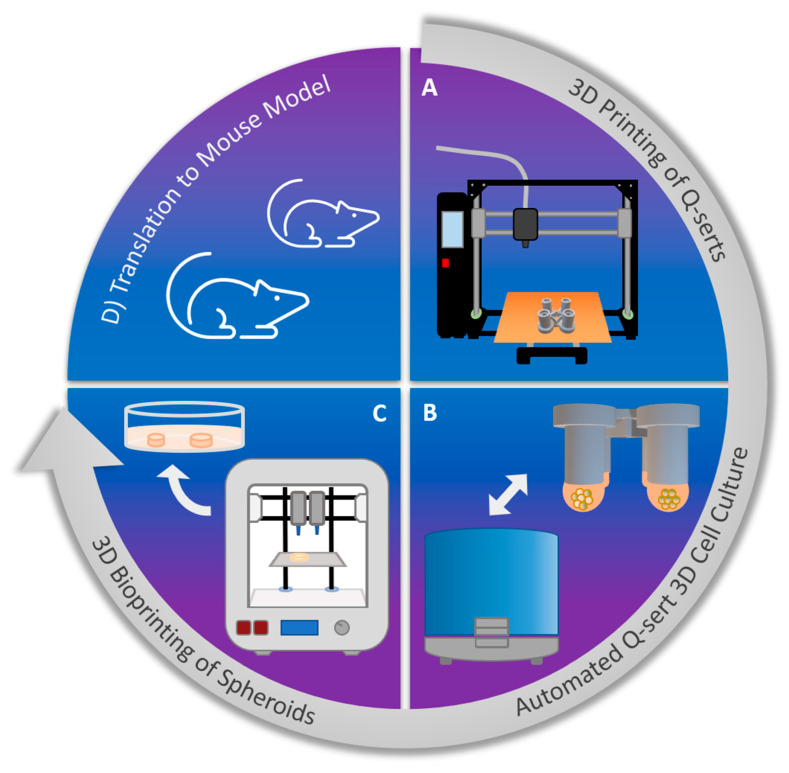
Workflow of the next generation of translational cancer research: (**A**) 3DP of devices for 3DCC using a readily available biocompatible polymer using a fused filament deposition (FFD) printer. (**B**) Culturing of cancer cells using hanging drop method in 3D printed devices (Q-serts), media change and transfer of spheroid using a standard pipetting robot. (**C**) 3DBP of spheroids using a common 3D bioprinter and a hydrogel bioink mimicking TME. (**D**) Extrapolation of findings from bioprinted spheroids’ cultures into rodent models and implantation of 3D printed spheroids into rodents.

In comparison to 2D cultures, 3D cultures are labor intensive and require highly specialized platforms, such as hanging drop plates for spheroid formation. Furthermore, the small size of the cellular constructs requires extensive technical skills and manhours and can lead to massive user-induced variability. By standardizing the fabrication of 3D culture systems and incorporating automation, such user errors can be diminished leading to improved workflow and output. It has already been shown that 3D models used in high-throughput (HT) analysis can help create meaningful data, which is translatable to animal models [23,24]. Cancer research can benefit from HT analytics and data gathering and drive personalized medicine through development of patient-specific tissue models. Toward this broader objective, in this work, we present a workflow for the next generation of translational cancer research (Figure 1), which applies 3D printing (3DP) and automation as central technologies to standardize 3DCC. With this workflow, the 3D tumor models can be realized facilely and in a standardized manner in the laboratory thus providing a bridge between in vitro and in vivo studies.

## 2. Results

### 2.1. 3D Printing and Sterilization of Q-Serts

**Figure 2:**
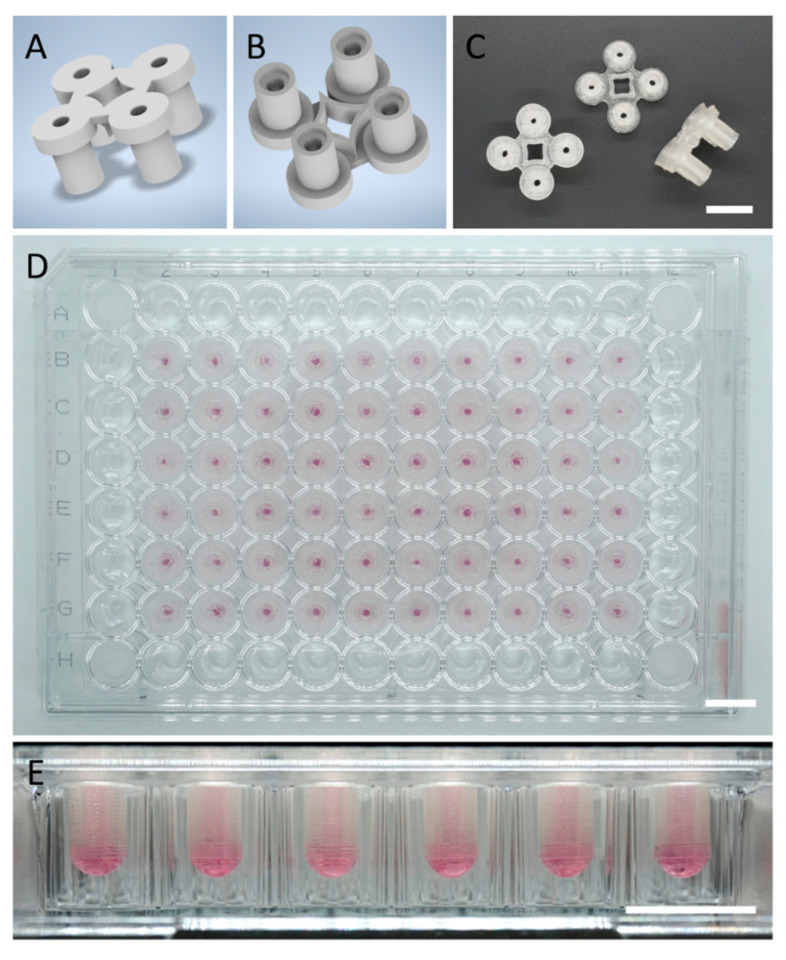
Design and application of Q-serts for 96-well plates. (**A**) Rendered top view of a Q-sert. (**B**) Rendered bottom view of a Q-sert. (**C**) Photographs of the top, bottom, side of Q-serts. (**D**) Fully loaded 96-well plate. (**E**) Drop formation. Scale bars (**C**–**E**) 10 mm.

The hanging drop technique was chosen as it is an established and widely accepted 3DCC method and easy to scale up. However, the limited commercial sources of hanging drop plates, combined with the high per-plate cost, is currently the bottleneck and an impediment to wider adoption of this culturing methodology in biomedical sciences. Sustainability is an important consideration in the development of new tools for biomedical research, as biomedical research generates a lot of waste that cannot be readily disposed of. Introducing biodegradable and compostable materials into fabrication of cell culture labware can significantly and positively contribute to the sustainability of biomedical research. With this goal in mind, we chose to apply PLA, as it represents the most widely used polymer derived from renewable sources, is biodegradable, and more importantly, cytocompatible. It possesses physical and chemical stability at physiological temperatures and pH for at least a few weeks [25]. PLA filaments were processed into Q-serts using FFD printing (Figure 2A–C). The design allows for easy “snap on” and stable placement inside a standard 96-well plate, facile pipetting, and observation of spheroids without the need for removal due to the channel configuration (Figure 2D). Thus, one can customize the 96-plate to have the desired number of Q-serts reducing waste and cost associated with experiments. Additionally, the design of the channels allow microscopy with focus-limited microscopes, enabling continuous monitoring of the spheroid formation. Prior to cell seeding, Q-serts were sterilized by ethanol and UV light, and the sterility of the Q-serts was confirmed by a negative PCR test for mycoplasma and the absence of any visual bacterial contamination. An image of a 96-well plate fully loaded with Q-serts is shown in Figure 2D along with the drop formation in Figure 2E. The Q-serts were stable over the course of the experiments (Figure 2E).

### 2.2. Culture of Spheroids in 3D Printed Q-Serts

**Figure 3:**
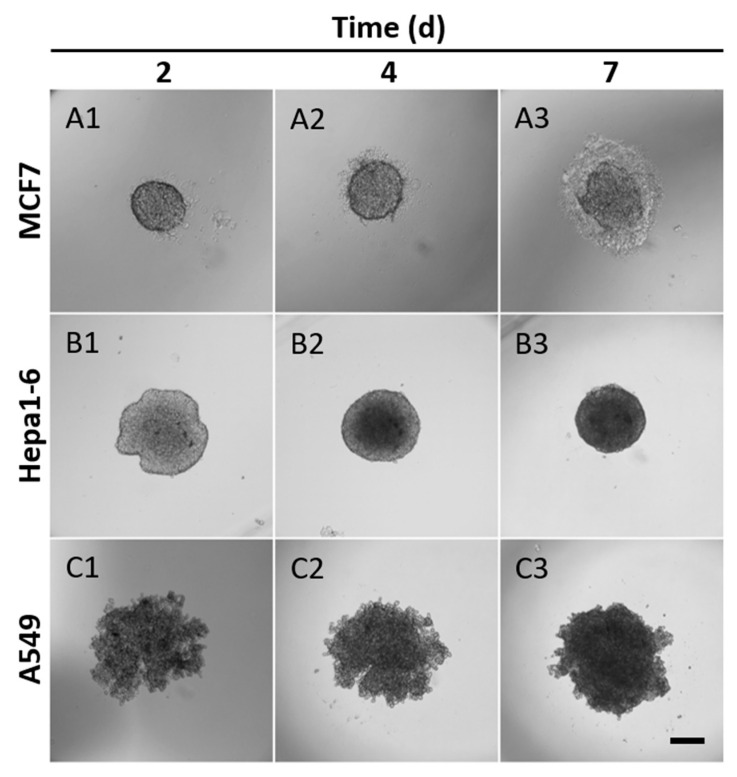
Spheroid formation in Q-serts over time. (**A1**–**A3**) MCF7, (**B1**–**B3**) Hepa1-6 and (**C1**–**C3**) A549 cells were used and monitored over the course of 7 days, 5000 cells seeded, scale bar 200 µm. 1: day 2, 2: day 4, 3: day 7.

In order to evaluate the suitability of the Q-serts, we monitored several cell lines (MCF7, Hepa1-6, and A549) with regard to their spheroid formation over time. In this setup, 5000 cells were used per hanging drop. All cell lines had aggregated in the center of the hanging drop by day 2, though the growth patterns differed between the cell lines (Figure 3). For MCF7, cells formed an oval shape in the beginning and grew bigger afterwards (Figure 3(A1–A3)). A semi-transparent corona of cells and secreted ECM gradually formed around the MCF7 spheroids and became obviously noticeable on day 7 (Figure 3(A1–A3)). Hepa1-6 cells initially formed as an irregular cell aggregation with an optically denser center (Figure 3(B1)). The aggregates condensed gradually and turned into a spherical shape (Figure 3(B2,B3)). In contrast, A549 assemblies were less spherical with a rough edge at first and then became denser aggregates with subtle change in diameter (Figure 3(C1–C3)). After 7 days of culture, most of the cells within the spheroids were viable (Figure S1).

### 2.3. Automated Spheroid Culture Handling

**Figure 4:**
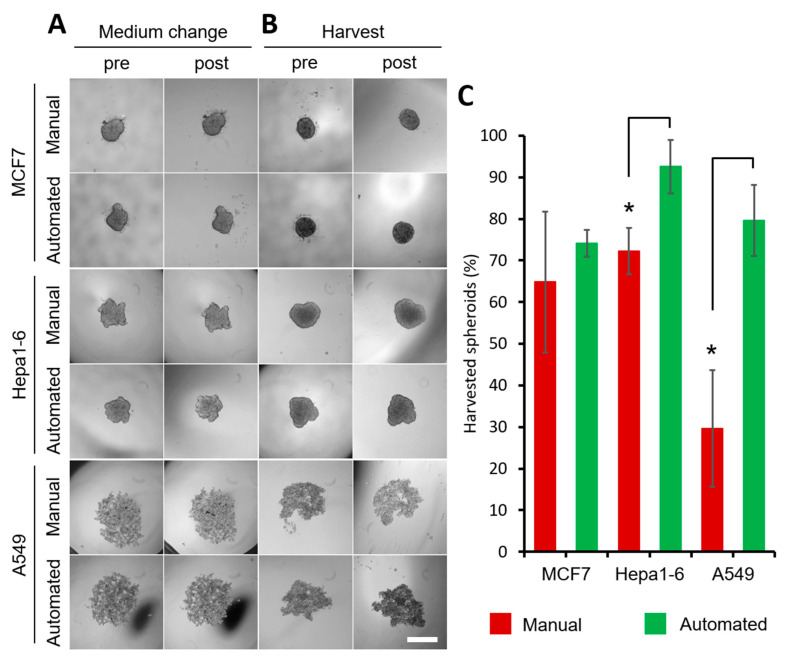
Evaluation of manual and automatic handling of hanging drop cell culture. (**A**) MCF7 and Hepa1-6 spheroids on day 2, A549 on day 3 before and after medium change. (**B**) MCF7, Hepa1-6 spheroids harvested on day 3, A549 spheroids harvested on day 5 before and after harvesting. 5000 cells were initially seeded, scale bar 500 µm. (**C**) Efficiency of spheroid harvest with automated and manual handling, n = 3 with 18 spheroids per n, * *p* < 0.05 significant differences between automated and manual harvest, red: manual harvest, green: automated harvest.

One aspect of using cellular spheroids for gathering large data sets is strain imposed on the operator by the tedious and repetitive nature of spheroid harvest, transfer and media change. Therefore, spheroid handling is a task well-suited for automation. The automation minimizes time and errors, and can help in standardizing the procedure and requires less manpower. It can be applied at several steps in the process, such as spheroid seeding, media change, spheroid harvest, and suspension of spheroids in the bioink. Here, we chose to address the media change (Figure 4A) and spheroid harvest (Figure 4B,C) through automation by deploying a liquid handling robot. The process is short, highly reproducible, and resource-efficient, while retaining the shape of the spheroids (Figure 4, Supplementary Video S1). The liquid handling aspect was qualified with respect to the integrity of spheroids and the success of harvest by comparing automated and manual handling. Five thousand cells were used per drop and 3 different cell lines (MCF7, Hepa1-6, A549) were investigated. On the second day, for MCF7 and Hepa1-6, the spheroid shape was preserved and no changes in morphology were observed after changing the medium (Figure 4A). A549 cells took longer to attach to each other, but the medium change could be carried out automatically as of day 3 without disturbing the spheroid formation. No cells were detected in the aspirated medium. For our workflow, cultured spheroids must be collected and mixed with a bioink. Therefore, an easy collection is desirable. Instead of pipetting spheroids through to the well bottom and collecting them, we successfully aspirated them using both manual and automated setups (Figure 4B). The automated harvest was highly reproducible and superior with 74.07 ± 2.62% (MCF7), 92.59 ± 6.42% (Hepa1-6) and 79.63 ± 8.49% (A549) efficiency versus 64.81 ± 13.86% (MCF7), 72.22 ± 5.56% (Hepa1-6) and 29.62 ± 13.98% (A549) for manual harvesting, with spheroids possessing the same gross morphological traits after automated and manual handling (Figure 4B,C). Due to the fragility of A549 spheroids in the early days, the earliest day of harvest was day 5. For Hepa1-6 and A549 cells, the automated harvest efficiency was significantly higher compared to the manual group.

### 2.4. Comparison of the Culture of STEMs in Q-Serts versus Commercial Systems

STEMs is a multicellular spheroid system comprising of lung adenocarcinoma epithelial cells, human marrow-derived mesenchymal cells, and micro vascular endothelial cells previously described from our laboratory, and it captures the cellular heterogeneity and cellular compartments observed in progressing tumor environments [9]. The unique attribute of the STEMs is its ability to recapitulate important epithelial solid tumor traits such as necrotic core and drug resistant phenotype.

**Figure 5:**
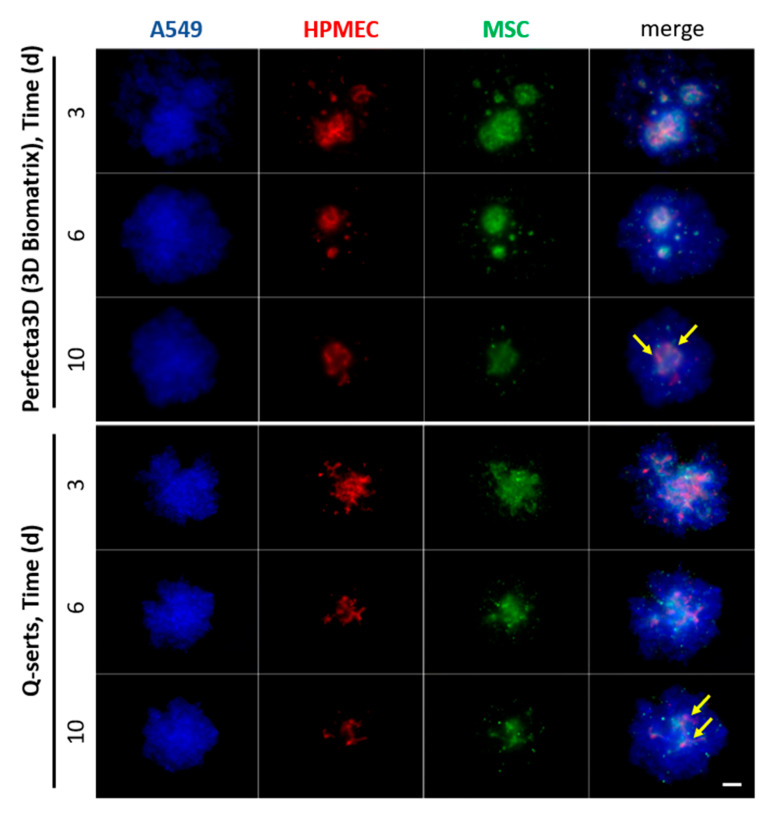
Time course of parallel culture of STEM spheroids in different hanging drop formats: 3D Biomatrix plate vs. Q-serts. A mixture of 25,000 cells (A549/HPMEC/MSC in ratio 5:3:2) was initially seeded. Imaging was done on days 3, 6, 10. Yellow arrows indicate central aggregates of HPMEC and MSCs. Scale bar 200 µm.

**Figure 6:**
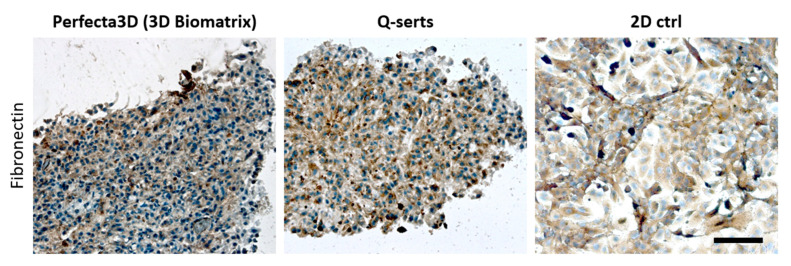
Immunohistochemical staining for fibronectin expressed in STEM spheroids grown in different hanging drop formats. 2D triculture served as positive control. Areas of fibronectin is denoted by brown stained regions. Hematoxylin counterstain was used for cell nuclei (blue). Scale bar 100 µm.

In order to differentiate between various cell populations, STEMs were prepared using fluorescently labeled cells (Table 1). The cells maintained their phenotypes after transduction, which was confirmed by their characteristic cell markers respectively, namely, pan-keratin for A549, CD31 for HPMEC and CD105 for MSC (Figure S2). A total of 25,000 cells (A549/HPMEC/MSC in ratio 5:3:2) were suspended and placed in the Q-serts and a commercial hanging drop device and yielded stable spheroids after 6 days in both cases (Figure 5). Both multicellular spheroids showed similar cellular organization as HPMEC and MSCs tended to aggregate in central parts of the spheroids, forming a core (Figure 5). Moreover, comparable sizes of the spheroids were formed during culture (Figure 5). Beyond this, we further evaluated the expression of fibronectin in the STEMs cultured in Q-serts and a commercially available system (Perfecta 3D), as fibronectin has been well known as a key protein associated with progression and malignancy of human lung adenocarcinoma [26,27,28]. Both STEMs cultured in Perfecta 3D and Q-serts demonstrated similar patterns of condensed cell aggregate with dark brown area wrapping cell nuclei, while in the 2D culture, fibronectin-positive (brown) area was more dispersed with more blank space (Figure 6). Thus, STEMs maintained their phenotype in Q-serts compared to the commercial system.

### 2.5. Rheology of Tumor-ECM Mimic Bioink

**Figure 7:**
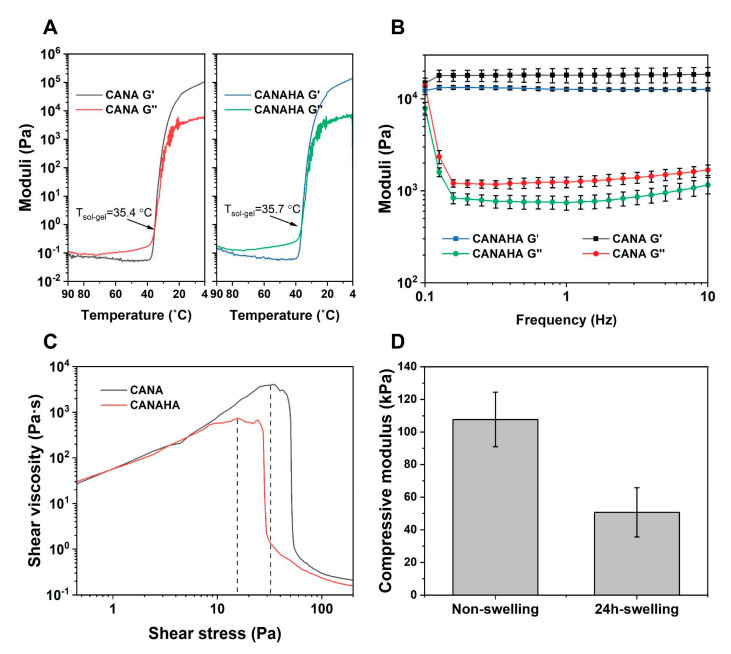
Rheological characterization of CANAHA bioinks. (**A**) Storage modulus (G′) and loss modulus (G″) of bioinks as a function of temperature. (**B**) Storage and loss moduli of bioinks at printing temperature (37 °C) under oscillatory frequency sweep from 0.1 Hz to 10 Hz. (**C**) Yield stress of bioinks at 37 °C. (**D**) Compressive moduli of molded CANAHA discs before and after swelling.

The development of bioinks for 3DBP is of critical importance when recreating an environment for cells. Three aspects were taken into consideration: (1) Mimicking the ECM and tissue environment; (2) proper rheological properties for printing, and (3) the stability of structures post-printing. We chose microextrusion-based 3DBP for this work, as it has the potential in building tall and complex structures. Built on our previous efforts in exploration in CA-based bioinks, the bioink used in this study was developed based on CA, since CA-based bioinks possess excellent printability with high cell viability, and inertness that allows further modification [16,17]. As such, in addition to the bioinks composed of CA and NA, HA was added in amounts as published for lung carcinomas to the bioink to better mimic the ECM of human lung carcinoma [29]. HA has been considered an important component in natural ECM in tissues like lungs and is one of the indices for disease progress [29]. Since the introduction of a new polymer has the potential to change the rheological behaviors of the composite, it necessitates rheological characterization. Therefore, a series of rheological tests to compare the CA-based bioink (CANA) and HA-added bioink (CANAHA) were carried out. As the gelling behavior is significant for extrusion-based printing, both bioinks were evaluated with regards to their storage (G′) and loss moduli (G″) over a temperature sweep. Upon addition of HA, no drastic changes in the gelling behavior were observed (Figure 7A) with gelling temperatures at 35.7 °C (±0.25 °C) versus 35.4 °C (±0.43 °C) for CANA bioink.

To further explore the properties during printing, a frequency sweep test and shear stress sweep test were carried out. It should be noted that all the samples were pre-treated as in actual printing. As shown in Figure 7B, CANAHA exhibited less viscosity than CANA bioink, which resulted in more fluidity, presenting lower yield stress (Figure 7C). In summary, it was observed that the addition of HA did not influence the general rheological behavior of the bioink. Furthermore, the stiffness of CANAHA bioink was measured using compressive tests to estimate the mechanical properties of the 3D printed matrix during culturing. Consistent with previous results, the compressive modulus dropped upon culturing from 107 ± 16.75 kPa to 50.73 ± 15.09 kPa (Figure 7D) [16].

### 2.6. 3D Bioprinting of Spheroids

**Figure 8:**
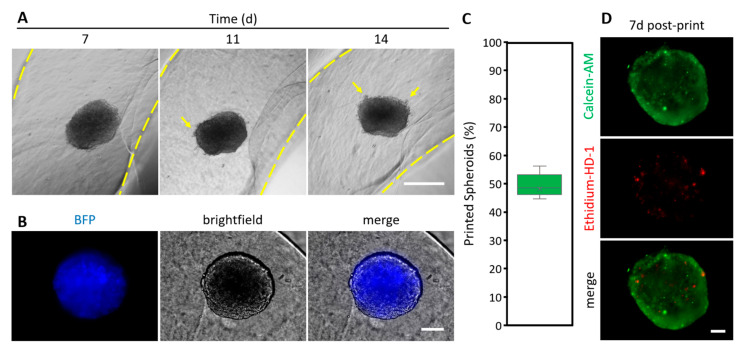
Printed spheroids in CANAHA bioink. (**A**) Time course of A549-BFP spheroid culture (yellow arrows indicate outgrowth of cells; space in yellow dashed lines indicates printed gel), scale bar 1000 µm. (**B**) Fluorescence microscopy image of A549-BFP spheroid 7 d after printing, scale bar 100 µm. (**C**) Rate of printed spheroids, n = 7 prints with 780 spheroids in total. (**D**) Calcein-AM and ethidium-HD-1 staining (live and dead assay) of bioprinted A549 spheroids 7 d after printing, scale bar 100 µm.

As a next step, we undertook the printing of the 3DCC constructs in a defined physical environment using microextrusion 3DBP. A549 spheroids (initially 5000 cells seeded) were harvested at day 7 and mixed with the CANAHA bioink for 3DBP. The reason for choosing A549 spheroids is that they appeared more fragile during handling compared to MCF7 or Hepa1-6 spheroids. Thus, by printing A549 spheroids, an upper limit of printability was set. The spheroids-laden bioink was printed successfully as a pre-designed ring design while retaining the shape of spheroids (Figure 8). During the following 7 days of culture, it was found that some cells were migrating from the spheroid and gradually dispersing into the surrounding hydrogel (Figure 8A), which suggests that the incorporation of the HA provides more tumor-ECM-like attributes to the CANA bioink environment. Since cells constitutively express fluorescent proteins, tracking of cells during printed spheroid culture was possible (Figure 8B), and this could be valuable in following changes to cells after, e.g., chemical or physical stimuli. In a series of 7 printing experiments, over 500 spheroids (n = 780) were harvested and mixed into a bioink to have 7 different spheroid-laden bioink cartridges ready for printing. In this series, 47.44 (±19.89) % of the harvested spheroids were successfully printed (Figure 8C). The printing efficiency of most attempts lies in the narrow interquartile range of the median (48.46%), with 44.62% as 1st quartile and 56.15% as 3rd quartile, which makes the process reproducible. Due to the small volume of bioink used per print (2.5 mL with an average of 130 spheroids), a rather high rate of spheroids/ink became stuck at the cartridge walls and could not be extruded. An example of a printed structure containing a spheroid is shown in Figure S3. Since the survival of the cells within the spheroids is an important criterion for a successful printing and the culture of a printed construct, the viability of cells was assessed 7 days after printing (Figure 8D). A spheroid that was not printed after 7 days of cultivation served as a control (Figure S1). No appreciable drop in cellular viability was observed in spheroids in the 3D printed environments after 14 days (7 days after printing) of culture (Figure 8D), especially not on the edges, where shear stress is exerted on the cells during the print.

## 3. Discussion

Biomedical research has significantly benefited from advances in engineering and computational sciences. Big data, enabled by HT screening and large-scale processing, has driven bioinformatics and provided new insights into epidemiology, drug research, and risk factors associated with disease progression [24,30,31,32]. A bottleneck in realizing the fruits of big-data analysis is gathering of immense quantities of data that is relevant to in vivo disease progression in an efficient manner with regards to human manpower, time, and resources. The confluence of emerging technologies-microfluidics, soft robotics, liquid handling robots, 3DP, single cell sequencing (omics)–with the internet of things (IoT) provides a unique landscape for gathering, dissemination, and analysis of data to further cancer research. In data analysis, the standardization of experimental set ups and data gathering is necessary to draw global, meaningful conclusions.

In this study, we have exploited 3DP to lay the groundwork for a standardized workflow for developing and utilizing in vitro systems to mimic tumorigenesis and understanding the role of tumor ECM. Cost and access to technology platforms serve as a primary impediment for the adoption of cutting-edge workflow across geographical and economic spectrums. As a first step towards addressing these issues, we have leveraged rapidly emerging technologies that have achieved deep penetration into scientific environments, namely 3DP, 3DBP, and multi axis robotic systems, to implement a low-cost, high-performance platform for 3DCC. Recently, Zhao et al. reported a “hanging drop dripper”, which is a 3D printed array for spheroid preparation and harvesting through gravity into a culture plate [33]. Here we have fabricated highly customizable and adaptive inserts, Q-serts, for spheroid generation, which by their design can (1) be scaled to accommodate both small and large experiments, thereby reducing waste and (2) allow for real-time visualization of spheroid formation using low power microscopes with short working distance objectives through the slender channel. Additionally, the system described herein differs from Zhao et al., in that it is modularized and suitable for HT workflows, including automation, and can further be scaled to fit any regular labware formats [33]. No complicated setups, e.g., microfluidics, are needed to cultivate spheroids, as reported elsewhere [34]. The Q-serts were fabricated using PLA, since it is the most common material of choice for FFD because of its wide availability as filament. Additionally, PLA was chosen over polyethylene terephthalate in this study, due to its favorable environment footprint, as the latter is sourced from petroleum distillates and not readily recyclable. Furthermore, PLA has a history of use in cell contacting applications without adverse effects [25]. The Q-serts were reliably produced without the need for any post-treatment, such as annealing, and they maintained the shape and integrity through the duration of the experiments.

Reproducibility is an important prerequisite of large-scale data gathering strategies such as a HT screening, which is the bedrock of drug discovery [4,35,36]. In cancer drug screening, automation of tumor cell spheroid culture can reduce variabilities attributed to human error in addition to saving time and resources. As illustrated in Supplementary Video S1, the aspiration of media, which is a tedious and repetitive task and can result in loss of spheroid if not done properly, is easily implemented in the Q-serts and can be accomplished using a generic liquid-handling robot that is commonly used with PCR setups.

In addition to the hanging drop method, tumor spheroids can be produced using centrifugation, forced floating, sedimentation and aggregation in geometrically confined low-adherence surfaces, and fluidics. Besides drug screening, tumor spheroids are routinely used to study tumor-associated events such as migration and invasion. Irrespective of the method of spheroid production for such studies presenting spheroids with a well-defined extracellular environment can promote reliable and predictable outcomes with regards to cellular organization and phenotype. Furthermore, tumor-associated events are best recapitulated in an environment that mimics the tumor. Similar considerations apply to studying organoids, as the organization of stem cells into organ-specific structures can be driven by geometry and mechanical considerations by “function follows form”. Since 3DBP can be scaled up, standardized, and integrated in an automated workflow, 3DBP of spheroids is perfectly suited to engineer bespoke tumor and organ models as it provides a means of introducing cells into complex biophysical environments by printing in a bioink that mimics important attributes of the tumor environment such as basement membrane, a vascular barrier, stiffness, and soluble signals. Automated handling of spheroids when combined with the technical workflow and the bioink developed in this study present a good starting point for incorporating 3DBP in cancer research.

In microextrusion 3DBP, which is the most common bioprinting platform, shear stress-induced damage to cells during printing is a concern [37,38,39], and here, the rheological properties of the bioinks play an important role as they define the printing parameters. There are two key considerations in designing bioinks for encapsulation of tumor spheroids, namely, appropriate rheological properties for printing and emulating the biophysical aspects of a tumor. HA, a highly water-soluble polysaccharide is ubiquitous in a cancer environment and plays an important role in hydration and cell-signaling through its cognate receptor CD44, a transmembrane glycoprotein that is expressed by cancer cells and cancer stems cells [40], and therefore can also function as a cell anchoring motif. Modification of the CANA bioink with HA yielded a bioink with lower yield stress (lower viscosity) and this translated into a low extrusion pressure. In contrast to literature reports, where shear stress induced loss in cell viability during printing, especially where in outer cell layers of spheroids was observed [39], using this bioink produced favorable outcomes with regards to cell viability even though a 4-fold higher number of cells were employed during the printing. Since a very limited number of studies have shown that the microextrusion-based 3DBP of spheroids can be done [39,41], the outcome of this study showing that the spheroids could cope with the shear stress in the bioink during extrusion is a notable step. Since the spheroids used for bioprinting were rather small and did not show zonal organization of cells from edge to center (proliferation, quiescent, necrotic zone) [8], future efforts should focus on larger and more physiologically relevant spheroids, such as STEMs [9]. However, increasing spheroid size can further exasperate the aggregation and sticking of the spheroid to the syringe wall during printing. Potential solutions to overcome this scenario and increase printing efficiency would involve use of a wiper piston to ensure the spheroids are fully and evenly extruded or a microfluidic-controlled printing head that enables co-printing of bioink and spheroids delivered through two disparate channels. If a certain number of bioprinted spheroids is desired, one should keep in mind the harvesting efficiency (varies by cell line) as well as the bioprinting efficiency (ca. 50%) to compensate for spheroid loss. In mimicking tissue environments, the mechanical properties of the bioink, in addition to the biology (ECM, soluble signals), needs to be taken into consideration as well. As an example, to study lung cancer formation or metastasis to the lung, emulating lung mechanics would be valuable. The modulus of the CANAHA bioink in its swollen state is about 10 times higher than that reported for lung tissue [42,43]. Therefore, strategies to lower the modulus while maintaining printability and designing constructs to impose physiological stress, i.e., cyclic loading need to be developed.

In summary, we have successfully developed a reproducible manufacturing process for the fabrication of advanced hanging drop inserts (Q-serts) using FFD printing, and demonstrated that Q-serts can be used to create multicellular, TME-mimicking spheroids in an automated manner for HT workflow. The cultured spheroids were further printed into predesigned 3D constructs that can replicate the spatial relation between tumor and surrounding tissues. The use of easily procurable instrumentation and raw materials with a simple user interface enabled a standardized, reproducible, and sustainable workflow. We envision that the spheroid culturing system described here can lay the foundation for the development of a widely accepted platform for drug screening, investigation of metastasis events, and omics studies, facilitating translational cancer research.

## 4. Materials and Methods

### 4.1. Cell Culture

Cells were cultured at 37 °C under 5% CO_2_ in their respective medium listed in Table 1. For passaging, cells at 70–80% confluency were washed with Dulbecco’s PBS (DPBS, Gibco, Germany) and trypsinized (0.05% trypsin/0.02% EDTA) for 5 min or until the majority of the cells detached. MCF7, Hepa1-6 and A549 cells were provided by the BIOSS toolbox (Centre for Biological Signalling Studies, University of Freiburg). Human pulmonary microvascular endothelial cells (HPMEC) were acquired from PromoCell (Heildelberg, Germany) and human marrow-derived mesenchymal stem cells (MSC) were kindly provided by Dr. Andrea Barbero and were obtained from patients under consent in accordance to the regulation of the institution’s ethical committee (University Hospital Basel; ref. nr. of local ethical committee 78/07).

### 4.2. Spheroid Culture

Sterilized Q-serts were snapped onto a 96-well plate. To form the spheroids, a 35 µL drop of a respective cell suspension was pipetted and the cellular aggregates were allowed to settle for 2 days, after which a daily medium change of 5 µL (removal) and 6 µL (addition, +1 µL to account for evaporation) was applied. Spheroids were harvested by pipetting 75 µL DPBS through each drop-containing hole. The Q-serts were removed and the spheroids were directly used or aspirated and collected in a collection tube. For comparison, a commercially sourced hanging drop system (Perfecta 3D, 3D Biomatrix, Ann Arbor, MI, USA) was used and the spheroids were prepared and handled in an identical manner as for the Q-serts. Where indicated, a medium change of the spheroid cultures was carried out using a QIAgility pipetting robot (Qiagen, Hilden, Germany). Briefly, 10 µL of medium was aspirated per well and pipetted to a waste tube, after which 10 µL was dispensed per well from a fresh medium tube. For automated harvesting of spheroids, spheroids were aspirated from the Q-serts and transferred to a collection tube. A t-test was performed to calculate significant differences between automated and manual handled cells of a respective cell line (* *p* < 0.05). Spheroids were visualized using a Zeiss Observer A1 (Carl Zeiss, Oberkochen, Germany), a Zeiss Axio Observer Z1 (Carl Zeiss, Oberkochen, Germany) or an Echo Revolve 4K microscope (Echo, San Diego, CA, USA).

### 4.3. Printing of Hanging Drop Inserts (Q-Serts)

Polylactic acid (PLA) filament (2.85 mm; Filamentworld, Neu Ulm, Germany) was used to print Q-serts for 96-well plates on a LulzBot Mini (FAME 3D, Fargo, ND, USA). The printed structures were designed using Inventor Professional 2022 (Autodesk, San Francisco, CA, USA). For use in cell culture, the printed devices were sterilized in 70% ethanol and irradiated with UV light for 30 min in a laminar flow hood.

### 4.4. Synthesis of Carboxylated Agarose

In this study, carboxylated agarose (CA) with a low shear modulus (400 Pa at 1 Hz, 2 *w/v*% gel), i.e., a high degree of carboxylation was used. CA was synthesized as previously described [44]. In brief, 10 g of native agarose (NA) type 1 (GeneOn, Germany) was transferred into a three-necked round bottom flask, equipped with a mechanical stirrer and pH meter. The reaction vessel was heated up to 90 °C to dissolve the agarose and then cooled down to 0 °C in an ice bath under mechanical stirring. The reactor was then charged with 300 mg TEMPO (Abcr, Karlsruhe, Germany), 1.5 g NaBr (0.9 mmol), and 37.5 mL NaOCl (15% *v/v* solution) under vigorous stirring. The pH of the solution was adjusted to pH 10.8 throughout the duration of the reaction, and the degree of carboxylation was controlled by the addition of predetermined volumes of NaOH solution (0.5 M). At the end of the reaction, 1.5 g NaBH_4_ was added, and the solution was acidified to pH 8 and stirred for 1 h. The CA was precipitated by sequential addition of 150 g NaCl and 500 mL ethanol, and the solid was collected by vacuum filtration and extracted using ethanol. Residual ethanol was removed by extensive dialysis against water and the CA was obtained as a white solid upon lyophilization overnight. The degree of carboxylation was verified by the appearance of peaks associated with aliphatic carboxylic acid groups via NMR 300 MHz (13C: 180 ppm) (Bruker BioSpin, Ettlingen, Germany).

### 4.5. Bioink Preparation

The CANA bioink formulation previously described by Gu et al. [16] was used as the basis for the development of a TME-mimic bioink (TME-Bioink). Briefly, lyophilized CA (95 mg) and NA (5 mg) were added into 1 mL DPBS and the mixture was heated up to 95 °C until a clear solution was obtained to yield a bioink composed of 10% *w/v* (9.5% CA + 0.5% NA) solids (CA + NA). The hot CANA solution was then filtered through a 0.45 µm syringe filter. HA (molecular weight 110 kDa) solution was prepared by dissolving 8 mg HA powder (Lot 10926-BA, Genzyme, Boston, MA, USA) in 1 mL DPBS, and the HA solution was filtered through a 0.22 µm syringe filter. For preparing CANAHA bioink, hot CANA bioink was first cooled to 45 °C and held at the temperature for 10 min to equilibrate the system, and then 10 µL HA solution was mixed with 1 mL CANA solution.

### 4.6. Rheology

A Kinexus Pro+ rotary rheometer (Malvern Instruments, Malvern, UK) was used for rheological assessments with a cone and plate assembly comprising an upper 4 cone plate 40 mm in diameter. Samples for rheological testing were prepared as follows: The sample was first heated to 95 °C until a clear solution was obtained before transferring to the stage set to a desired temperature. For the thermal-dynamic rheological characterization, samples were loaded on the lower plate at 45 °C and maintained for 5 min to equilibrate. Then the samples were cooled down to the target temperature at a rate of 5 °C/min at a constant frequency of 0.1 Hz and a constant shear strain of 1%. For the frequency sweeping, samples were loaded on the lower plate at 37 °C, equilibrated for 5 min before a frequency sweep from 10 Hz to 0.1 Hz. The yield stress was determined by a shear stress ramping test, starting from 0 Pa and ending with 200 Pa.

For the compression tests, bioink was prepared in a 2 mL syringe in solution state, and cooled down in the fridge to form gel, and then kept at room temperature for 3 h to reach equilibrium. Samples were cut from the syringe into a 2 mm high disc with surgical scalpel. Non-swelling samples were tested instantly after cutting, and swelling samples were tested after 24 h incubation in DPBS in a 37 °C incubator. The compressive tests were performed on the Kinexus Pro+ rheometer with an upper plate 20 mm in diameter. The compression rate was set to 1 × 10^−3^ mm/s. The compression was terminated when the upper plate reached the prescribed gap or when the detected normal force reached 50 N.

### 4.7. Bioprinting

The CANAHA bioink was transferred to a heating block set at 42 °C for at least 10 min. A spheroid suspension of 100 µL per 0.9 mL bioink was added and the solution was gently vortexed to achieve a homogenous distribution and CANAHA composition as described. Subsequently, the bioink was loaded into the printer cartridge at 37 °C and incubated for 15 min before commencing the print. An Inkredible-2 3D printer (Cellink, Gothenburg, Sweden), with several custom modifications including a temperature-controlled nozzle heater and a water-cooled print bed, manufactured in-house by the machine workshop at the Institute for Macromolecular Chemistry at the University of Freiburg, was used to print ring structures through an 18G nozzle. The g-code was generated in Slic3r (GNU Affero General Public License) and modified in HeartWare (v2.1.6; Cellink, Gothenburg, Sweden). The print bed was set to 4 °C to ensure immediate gelation of the bioink after extrusion. The printed structures were immediately placed in the respective culture medium for further culture and analysis.

### 4.8. Histology and Live-Dead Staining

For histological staining, spheroids were collected and washed in DPBS, and fixed in 3.7% formaldehyde DPBS overnight, and then transferred to 30% sucrose for embedding into OCT medium for cryosectioning. Slices (6 µm thickness) were obtained using a Hyrax C20 (Carl Zeiss, Oberkochen, Germany) set at −26 °C. The staining was done according to the following immunohistochemistry procedure: Sections were washed in PBS and blocked with 2.5% goat serum, 0.1% Triton-X, 0.05% Tween20 in PBS, and then the samples were incubated with the primary antibody (Fibronectin, 1:300, Abcam; CD31, 1:100, Abcam; CD105 1:100, Santa Cruz Biotechnology; Pan-keratin, 1:100, Abcam) overnight and washed afterwards before counterstaining with hematoxylin. Control sections were processed without the primary antibody. Cultures grown in 2D were directly stained without the need for sectioning.

For live-dead staining (LIVE/DEAD Viability/Cytotoxicity Kit, Invitrogen, Waltham, MA, USA), spheroids were collected and washed twice with DPBS. DPBS was aspirated and cells were stained with 2 µM calcein AM (live) and 5 µM ethidium homodimer-1 (EthD-1, dead) DPBS solution for 30–60 min. After the incubation, cells were washed with DPBS once and imaged.

## Figures and Tables

**Table 1:** Cultured cell lines and culture conditions. FBS: fetal bovine serum, P/S: penicillin/streptomycin, FGF2: fibroblast growth factor 2, BFP: blue fluorescent protein, RFP: red fluorescent protein, GFP: green fluorescent protein.

Cell Line (Label)	Culture Medium	Additive	Nr. of Cells
Hepa1-6	DMEM	10% FBS	5000
MCF7	DMEM	10% FBS	5000
A549 (BFP)	DMEM	10% FBS	5/10,000
A549 (BFP), HPMEC (RFP), MSC (GFP)	DMEM, ECGM, alpha-MEM	10% FBS, 100 U/mL P/S 5% FBS, 50 U/mL P/S 10% FBS, 5 ng/mL FGF2, 1% P/S	25,000 (5:3:2 cell and medium ratio)

## Data Availability

Data is contained within the article or Supplementary Material.

## Supplementary Materials

The following supporting information can be downloaded at: https://www.mdpi.com/article/10.3390/ijms23158188/s1.

## References

[1] (2020). WHO Report on Cancer: Setting Priorities, Investing Wisely and Providing Care for All.

[2] Hanahan D., Weinberg R.A. (2011). Hallmarks of cancer: The next generation. Cell.

[3] Hanahan D. (2022). Hallmarks of Cancer: New Dimensions. Cancer Discov..

[4] Jensen C., Teng Y. (2020). Is It Time to Start Transitioning From 2D to 3D Cell Culture?. Front. Mol. Biosci..

[5] Jong B.K. (2005). Three-dimensional tissue culture models in cancer biology. Semin. Cancer Biol..

[6] Griffith L.G., Swartz M.A. (2006). Capturing complex 3D tissue physiology in vitro. Nat. Rev. Mol. Cell Biol..

[7] Cortesi M.M., Zamagni A., Arienti C., Pignatta S., Tesei A. (2020). Modeling neoplastic disease with spheroids and organoids. J. Hematol. Oncol..

[8] Lin R.Z., Chang H.Y. (2008). Recent advances in three-dimensional multicellular spheroid culture for biomedical research. Biotechnol. J..

[9] Lamichhane S.P., Arya N., Kohler E., Xiang S., Christensen J., Shastri V.P. (2016). Recapitulating epithelial tumor microenvironment in vitro using three dimensional tri-culture of human epithelial, endothelial, and mesenchymal cells. BMC Cancer.

[10] Panek M., Grabacka M., Pierzchalska M. (2018). The formation of intestinal organoids in a hanging drop culture. Cytotechnology.

[11] Riede J., Wollmann B.M., Molden E., Ingelman-Sundberg M. (2021). Primary human hepatocyte spheroids as an in vitro tool for investigating drug compounds with low clearance. Drug Metab. Dispos..

[12] Caliari S.R., Burdick J.A. (2016). A practical guide to hydrogels for cell culture. Nat. Methods.

[13] Tibbitt M.W., Anseth K.S. (2009). Hydrogels as extracellular matrix mimics for 3D cell culture. Biotechnol. Bioeng..

[14] Liu C., Lewin Mejia D., Chiang B., Luker K.E., Luker G.D. (2018). Hybrid collagen alginate hydrogel as a platform for 3D tumor spheroid invasion. Acta Biomater..

[15] Xu X., Gurski L.A., Zhang C., Harrington D.A., Farach-Carson M.C., Jia X. (2012). Recreating the tumor microenvironment in a bilayer, hyaluronic acid hydrogel construct for the growth of prostate cancer spheroids. Biomaterials.

[16] Gu Y., Schwarz B., Forget A., Barbero A., Martin I., Shastri V.P. (2020). Advanced Bioink for 3D Bioprinting of Complex Free-Standing Structures with High Stiffness. Bioengineering.

[17] Forget A., Blaeser A., Miessmer F., Köpf M., Campos D.F.D., Voelcker N.H., Blencowe A., Fischer H., Shastri V.P. (2017). Mechanically Tunable Bioink for 3D Bioprinting of Human Cells. Adv. Healthc. Mater..

[18] Ying G., Jiang N., Yu C., Zhang Y.S. (2018). Three-dimensional bioprinting of gelatin methacryloyl (GelMA). Bio-Des. Manuf..

[19] Ma L., Li Y., Wu Y., Yu M., Aazmi A., Gao L., Xue Q., Luo Y., Zhou H., Zhang B. (2020). 3D bioprinted hyaluronic acid-based cell-laden scaffold for brain microenvironment simulation. Bio-Des. Manuf..

[20] Zhang Y.S., Duchamp M., Oklu R., Ellisen L.W., Langer R., Khademhosseini A. (2016). Bioprinting the Cancer Microenvironment. ACS Biomater. Sci. Eng..

[21] Gu Y., Forget A., Shastri V.P. (2022). Biobridge: An Outlook on Translational Bioinks for 3D Bioprinting. Adv. Sci..

[22] Yuan Y. (2016). Spatial Heterogeneity in the Tumor Microenvironment. Cold Spring Harb. Perspect. Med..

[23] LaBarbera D.V., Reid B.G., Yoo B.H. (2012). The multicellular tumor spheroid model for high-throughput cancer drug discovery. Expert Opin. Drug Discov..

[24] Hongisto V., Jernström S., Fey V., Mpindi J.P., Kleivi Sahlberg K., Kallioniemi O., Perälä M. (2013). High-throughput 3D screening reveals differences in drug sensitivities between culture models of JIMT1 breast cancer cells. PLoS ONE.

[25] da Silva D., Kaduri M., Poley M., Adir O., Krinsky N., Shainsky-Roitman J., Schroeder A. (2018). Biocompatibility, biodegradation and excretion of polylactic acid (PLA) in medical implants and theranostic systems. Chem. Eng. J..

[26] Sethi T., Rintoul R.C., Moore S.M., MacKinnon A.C., Salter D., Choo C., Chilvers E.R., Dransfield I., Donnelly S.C., Strieter R. (1999). Extracellular matrix proteins protect small cell lung cancer cells against apoptosis: A mechanism for small cell lung cancer growth and drug resistance in vivo. Nat. Med..

[27] Han S., Sidell N., Roman J. (2005). Fibronectin stimulates human lung carcinoma cell proliferation by suppressing p21 gene expression via signals involving Erk and Rho kinase. Cancer Lett..

[28] Meng X.N., Jin Y., Yu Y., Bai J., Liu G.Y., Zhu J., Zhao Y.Z., Wang Z., Chen F., Lee K.Y. (2009). Characterisation of fibronectin-mediated FAK signalling pathways in lung cancer cell migration and invasion. Br. J. Cancer.

[29] Rangel M.P., de Sá V.K., Martins V., Martins J., Parra E.R., Mendes A., Andrade P.C., Reis R.M., Longatto-Filho A., Oliveira C.Z. (2015). Tissue hyaluronan expression, as reflected in the sputum of lung cancer patients, is an indicator of malignancy. Braz. J. Med. Biol. Res..

[30] Thomas R.K., Baker A.C., DeBiasi R.M., Winckler W., LaFramboise T., Lin W.M., Wang M., Feng W., Zander T., MacConnaill L.E. (2007). High-throughput oncogene mutation profiling in human cancer. Nat. Genet..

[31] Glas A.M., Floore A., Delahaye L.J.M.J., Witteveen A.T., Pover R.C.F., Bakx N., Lahti-Domenici J.S.T., Bruinsma T.J., Warmoes M.O., Bernards R. (2006). Converting a breast cancer microarray signature into a high-throughput diagnostic test. BMC Genomics.

[32] Yang C., Luo J., Polunas M., Bosnjak N., Dean Chueng S.-T., Chadwick M., Sabaawy H.E., Chester S.A., Lee K.-B., Lee H. (2020). 4D-Printed Transformable Tube Array for High-Throughput 3D Cell Culture and Histology. Adv. Mater..

[33] Zhao L., Xiu J., Liu Y., Zhang T., Pan W., Zheng X., Zhang X. (2019). A 3D Printed Hanging Drop Dripper for Tumor Spheroids Analysis without Recovery. Sci. Rep..

[34] Frey O., Misun P.M., Fluri D.A., Hengstler J.G., Hierlemann A. (2014). Reconfigurable microfluidic hanging drop network for multi-tissue interaction and analysis. Nat. Commun..

[35] Doulgkeroglou M.-N., Di Nubila A., Niessing B., König N., Schmitt R.H., Damen J., Szilvassy S.J., Chang W., Csontos L., Louis S. (2020). Automation, Monitoring, and Standardization of Cell Product Manufacturing. Front. Bioeng. Biotechnol..

[36] Han S.J., Kwon S., Kim K.S. (2021). Challenges of applying multicellular tumor spheroids in preclinical phase. Cancer Cell Int..

[37] Nair K., Gandhi M., Khalil S., Yan K.C., Marcolongo M., Barbee K., Sun W. (2009). Characterization of cell viability during bioprinting processes. Biotechnol. J..

[38] Boularaoui S., Al Hussein G., Khan K.A., Christoforou N., Stefanini C. (2020). An overview of extrusion-based bioprinting with a focus on induced shear stress and its effect on cell viability. Bioprinting.

[39] Horder H., Guaza Lasheras M., Grummel N., Nadernezhad A., Herbig J., Ergün S., Teßmar J., Groll J., Fabry B., Bauer-Kreisel P. (2021). Bioprinting and Differentiation of Adipose-Derived Stromal Cell Spheroids for a 3D Breast Cancer-Adipose Tissue Model. Cells.

[40] Misra S., Hascall V.C., Markwald R.R., Ghatak S. (2015). Interactions between Hyaluronan and Its Receptors (CD44, RHAMM) Regulate the Activities of Inflammation and Cancer. Front. Immunol..

[41] Swaminathan S., Hamid Q., Sun W., Clyne A.M. (2019). Bioprinting of 3D breast epithelial spheroids for human cancer models. Biofabrication.

[42] Barreiro Carpio M., Dabaghi M., Ungureanu J., Kolb M.R., Hirota J.A., Moran-Mirabal J.M. (2021). 3D Bioprinting Strategies, Challenges, and Opportunities to Model the Lung Tissue Microenvironment and Its Function. Front. Bioeng. Biotechnol..

[43] Polio S.R., Kundu A.N., Dougan C.E., Birch N.P., Aurian-Blajeni D.E., Schiffman J.D., Crosby A.J., Peyton S.R. (2018). Cross-platform mechanical characterization of lung tissue. PLoS ONE.

[44] Forget A., Christensen J., Lüdeke S., Kohler E., Tobias S., Matloubi M., Thomann R., Prasad V.S. (2013). Polysaccharide hydrogels with tunable stiffness and provasculogenic properties via α-helix to β-sheet switch in secondary structure. Proc. Natl. Acad. Sci. USA.

