# Supplementary material for: 3D Printed Solutions for Spheroid Engineering and Cancer Research
Source: Int J Mol Sci. 2022 Jul 25;23(15):8188. doi: 10.3390/ijms23158188 (PMC9331260; doi:10.3390/ijms23158188)
Supplement: Supplementary file 1 [file ijms-23-08188-s001.zip › ijms-1756795-R-supplementary_FINAL.pdf]

Supplementary material

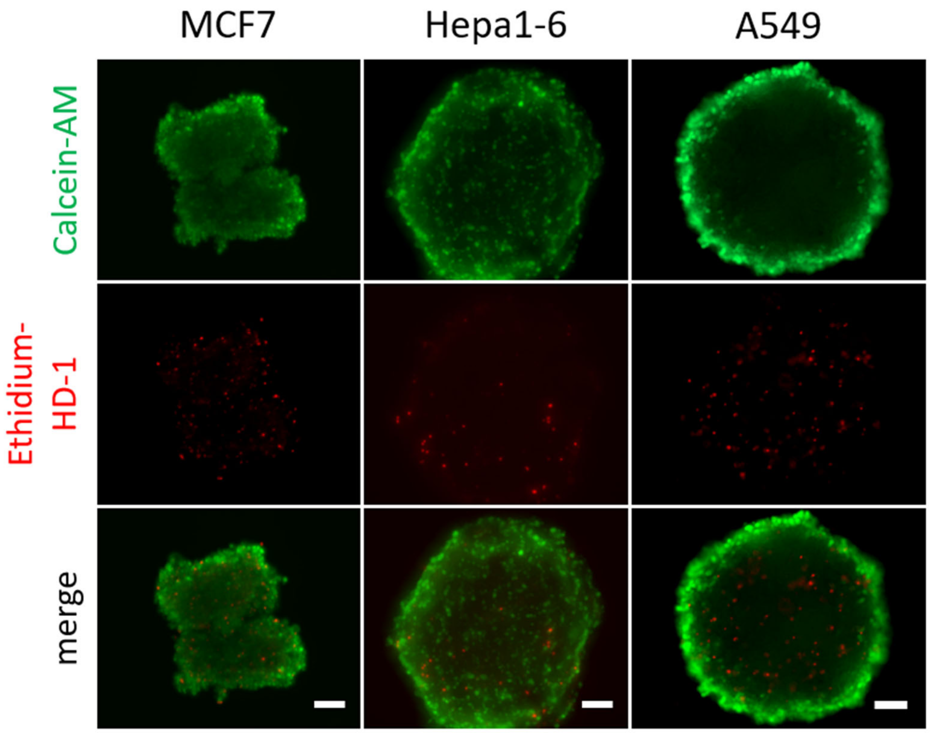

Figure S1. Calcein-AM and Ethidium-HD-1 staining as live and dead assay for MCF7, Hepa1-6, A549 spheroids. Images taken on day 7 (hanging drop) and 8 and 14 (printed A549). 5,000 cells were initially seeded. Scale bars: 100 μm.

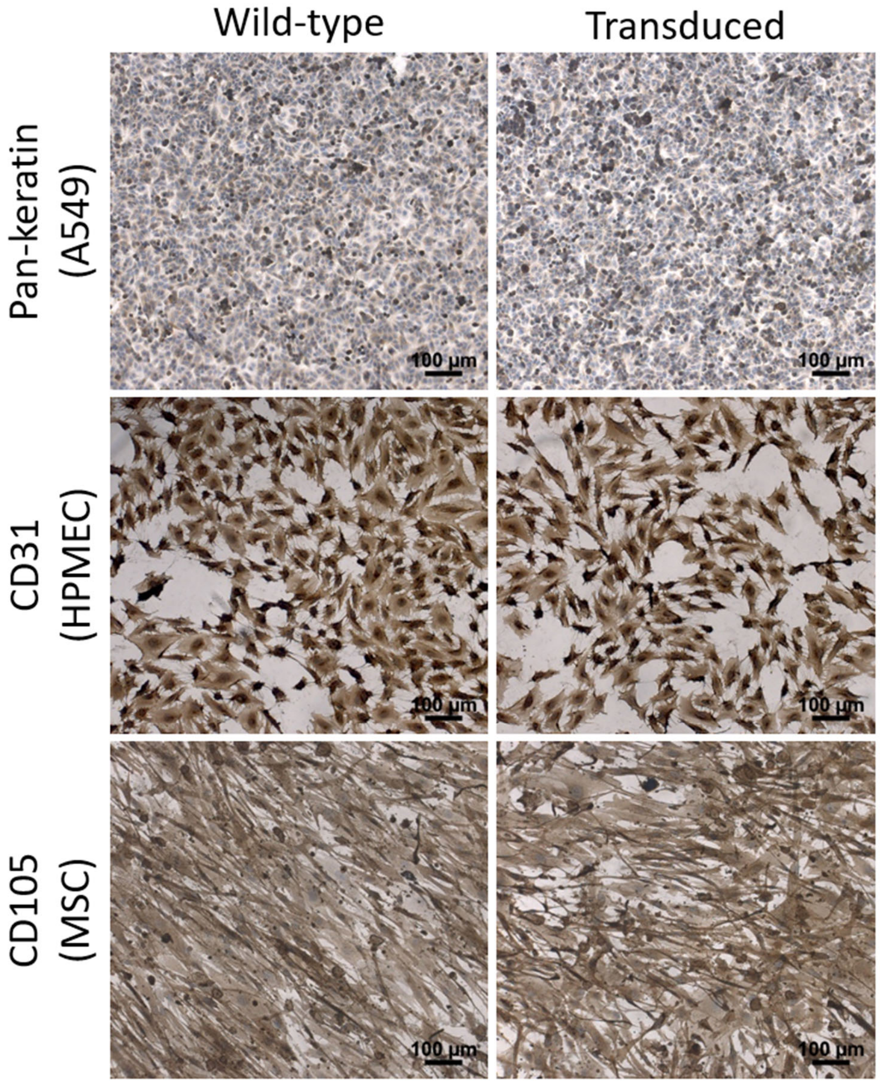

Figure S2. Control staining of wild-type and transduced cells. Pan-keratin staining for A549, CD31 for HPMEC, CD105 for MSC. Scale bars: 100 μm.

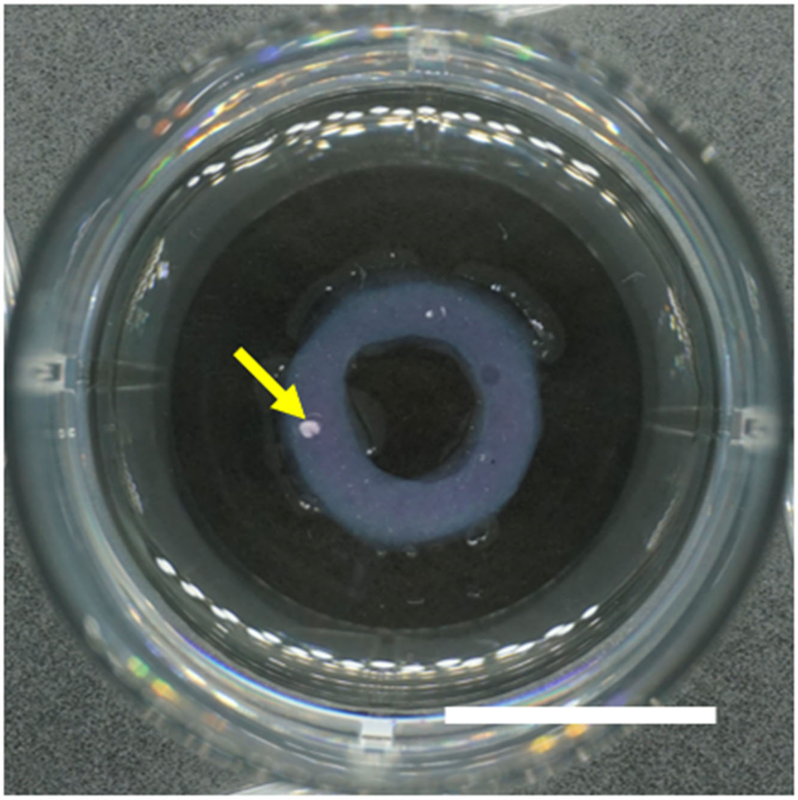

Figure S3. Bioprinted spheroid-containing CANAHA ring**.** Picture taken in a 12 well dish containing DPBS, after 14 d of regular culture. Yellow arrow indicates site of printed spheroid, scale bar 10 mm.
